# Dietary Zinc Glycine Supplementation Attenuates Aging-Driven Cochlear Hair Cell Oxidative Injury and Hearing Loss

**DOI:** 10.3390/nu18193121

**Published:** 2026-09-23

**Authors:** Xue Bai, Kai-Lang Zhou, Wen-Hui Hu, Li Fu, Sha-Sha Sun, Yi-Meng Yuan, Kai Xu, Yue-Hui Liu

**Affiliations:** Department of Otolaryngology, Head and Neck Surgery, The Second Affiliated Hospital of Nanchang University, Jiangxi Medical College, Nanchang University, Nanchang 330006, China; 360014240004@email.ncu.edu.cn (X.B.);

**Keywords:** micronutrients, zinc glycinate, age-related hearing loss, MTF-1, cGAS-STING pathway

## Abstract

**Background:** Micronutrient imbalance is now recognized as a modifiable risk factor driving the progression of age-related hearing loss (ARHL). Zinc, an indispensable dietary trace nutrient, acts as a structural cofactor and transcriptional regulator of a broad panel of antioxidant enzymes to sustain systemic and cellular antioxidant defense capacity. Nevertheless, the therapeutic value of zinc nutritional supplementation and its precise molecular mechanisms against age-dependent cochlear degeneration remain largely uncharacterized. **Methods:** We established aging mouse models to observe the phenotypic changes caused by disrupted cochlear zinc nutritional homeostasis, and applied oral supplementation of bioavailable zinc glycinate to intervene in ARHL. In vitro D-galactose (D-gal) stimulation was adopted to induce cellular oxidative damage, followed by a series of mechanistic assays to detect the expression of core antioxidant molecules and determine mitochondrial function. **Results:** Disrupted cochlear zinc nutritional homeostasis induced progressive hearing loss and severe hair cell degeneration in aging mice. Conversely, oral zinc glycinate supplementation significantly relieved age-related hearing decline and reduced oxidative injury of cochlear hair cells. As a nutritional intervention, zinc glycinate significantly elevates the levels of key intracellular antioxidants (SOD, GSH, and GPX4) and rescues mitochondrial dysfunction induced by D-galactose triggered reactive oxygen species accumulation. Moreover, the protective effect of zinc glycinate may be associated with the upregulation of metal-responsive transcription factor 1 (MTF-1), which in turn suppresses the D-galactose-induced hyperactivation of the pro-inflammatory cGAS-STING pathway. **Conclusions:** Dietary zinc glycinate supplementation offers potential protection against cochlear aging. This auditory benefit may involve MTF-1 upregulation and inhibition of the cGAS-STING pathway. Collectively, these findings provide preclinical evidence supporting further investigation of zinc glycinate as a potential nutritional intervention for ARHL.

## 1. Introduction

With the acceleration of global population aging, presbycusis, also known as age-related hearing loss (ARHL), has become the most common chronic sensorineural degenerative disorder among the elderly worldwide [1]. According to data from the World Health Organization, approximately one-third of individuals over 65 years of age experience moderate or greater hearing loss, and the large number of elderly people affected by this condition places a heavy economic burden on families and public health systems [2]. ARHL is a progressive sensorineural hearing loss driven primarily by aging, resulting from the interplay of multiple factors including genetic susceptibility, environmental exposure, and nutritional imbalance [3]. Accumulating epidemiological evidence further indicates that long-term untreated hearing loss is closely associated with cognitive decline, depression, and an increased risk of dementia in the elderly [4,5]. Currently, existing clinical interventions, such as hearing aids and cochlear implants, can only partially compensate for auditory function and cannot fundamentally repair pathological damage [6]. Therefore, there is an urgent need to explore effective molecular targets and targeted protective agents that can delay cochlear aging, thereby providing new strategies for the prevention and treatment of ARHL.

In recent years, nutritional imbalance and micronutrient insufficiency have emerged as modifiable non-genetic risk factors for ARHL, representing a promising, translational research direction within nutritional geroscience to complement existing pathogenic frameworks [7,8]. Deficiencies in essential dietary nutrients can directly or indirectly exacerbate ARHL onset and progression by suppressing inner ear antioxidant enzyme activity, disrupting mitochondrial energy metabolism and dysregulating cytoprotective signaling pathways in auditory cells [9,10]. Zinc is an indispensable dietary trace micronutrient for human physiological homeostasis, functioning as a structural cofactor and transcriptional regulator for a broad spectrum of core antioxidant enzymes to maintain cellular redox equilibrium [11,12]. Under physiological zinc-replete nutritional status, zinc stabilizes the catalytic activity of superoxide dismutase and glutathione reductase, elevates intracellular glutathione (GSH) reserves, and sustains global redox balance within sensory cells [13]. Intracellular labile Zn^2+^ concentrations are tightly governed by a multi-layered zinc homeostatic network comprising zinc transporters, cytoplasmic metal-buffering proteins and organellar zinc sequestration machinery, which coordinate transmembrane zinc uptake, rapid cytoplasmic buffering and compartmentalized storage [14,15]. Two major zinc transporter superfamilies dominate this regulatory system: ZIP proteins mediate extracellular Zn^2+^ influx into the cytoplasm, while ZnT proteins facilitate cytoplasmic Zn^2+^ efflux across cellular membranes [16,17]. Moreover, metal-responsive transcription factor 1 (MTF-1) senses fluctuations in cytosolic free Zn^2+^ levels via its N-terminal zinc finger domains and initiates transcriptional upregulation of downstream antioxidant and cytoprotective genes upon adequate zinc supply [12]. Prior epidemiological and preclinical studies have linked dietary zinc deficiency to multiple age-related neurodegenerative pathologies and aggravated hearing impairment [18]. However, the otoprotective efficacy of zinc nutritional supplementation, the specific antioxidant mechanisms mediated by bioavailable zinc glycinate, and the functional crosstalk between MTF-1 and oxidative-inflammatory signaling in ARHL have not yet been systematically characterized. Therefore, we investigated the protective effects of modulating cochlear zinc homeostasis on auditory oxidative damage and the underlying molecular mechanisms.

In the present work, we established naturally aged mouse models and D-gal-induced oxidative damage models of cochlear hair cells to delineate the auditory protective benefits of zinc glycinate as a nutritional supplement against age-related hearing loss and dissect its underlying molecular cascade. In vivo animal assays first verified that disrupted cochlear zinc nutritional homeostasis drives progressive hearing impairment and severe oxidative degeneration of cochlear hair cells in aged mice. In contrast, oral supplementation with zinc glycinate, a readily absorbable nutritional form of zinc, partially ameliorated ARHL and alleviated hair cell damage induced by reactive oxygen species (ROS). Further mechanistic investigation revealed that the protective effect of zinc glycinate may be mediated through upregulation of MTF-1 expression, which in turn suppressed the aberrant activation of the D-galactose-induced pro-inflammatory cGAS-STING pathway. Collectively, our findings demonstrate that dietary zinc glycinate supplementation significantly enhances the expression of cochlear antioxidant enzymes, effectively exerts anti-aging protection, and attenuates age-related oxidative damage to cochlear hair cells. This study delineates a novel micronutrient regulatory axis involved in cochlear aging and provides evidence at both the cellular and animal model levels, supporting zinc glycinate as a promising nutritional intervention candidate for the prevention and adjunctive treatment of ARHL.

## 2. Materials and Methods

### 2.1. Mouse Model

C57BL/6J mice from Nanchang University’s Animal Research Center were used for all in vivo tests. Neonatal littermates were obtained via timed adult mating. All mice were housed in a specific pathogen-free (SPF) laboratory animal facility, under a 12 h light/12 h dark circadian cycle. Constant ambient temperature was maintained at 23 ± 1 °C, and relative humidity was controlled at 60% ± 5%. Eight-week-old male C57BL/6J mice were acclimated to the laboratory environment for one week, and then randomly assigned to different experimental groups. Control mice were fed a standard breeding diet, whereas zinc-deficient mice received a custom-made zinc-deficient diet. All mice had free access to deionized water throughout the experiments. In the zinc-supplemented mouse model, zinc glycinate was administered at a final concentration of 120 mg/kg. All animal operations complied with the NIH laboratory animal care guidelines and were approved by Nanchang University’s animal ethics committee (Approval No. NCULAE-20241032026). At the start of grouping, the body weight of all mice was confined to the range of 25 ± 2 g. We minimized animal quantity and alleviated pain during all experimental manipulations. All sample labels were replaced by a uniformly encoded serial number before the test. During the data collection and analysis process, no grouping information was obtained.

### 2.2. Zinc Ion Level Detection

At the end of each dietary intervention cycle, all animal models were deeply anesthetized with intraperitoneally injected pentobarbital prior to tissue harvesting. After stable anesthesia was achieved, whole blood was collected from the posterior vena cava. Blood samples were centrifuged at 13,200× *g* for 10 min to extract serum, which was stored at −20 °C for subsequent biochemical detection. Following blood collection, rapid transection of the thoracic artery was performed to complete euthanasia. Complete cochlear tissues were then carefully isolated. A commercial colorimetric zinc quantification kit was used to determine zinc concentrations in both serum and cochlear specimens, with all operations conducted in compliance with the manufacturer’s specifications. All acquired zinc level data were compiled for subsequent statistical analysis.

### 2.3. Auditory Function Assessment

ABR and DPOAE recordings were executed referencing established measurement workflows from prior work [5,19]. Prior to hearing testing, deep sedation was induced in all mice via intraperitoneal co-administration of ketamine (120 mg/kg) and chlorpromazine (20 mg/kg), after which animals were transferred into a sound-insulated testing compartment. Subcutaneous electrode placement was implemented: one recording electrode was positioned beneath the scalp, while a reference electrode was attached to the skin surface above the tested cochlea. The body temperature was kept by using a heating pad. Every mouse underwent a single round of testing, with each subject counted as a separate biological replicate. Tone-burst stimuli spanning 8, 16, 24, 32 and 40 kHz were applied for ABR acquisition, and 1024 signal sweeps were averaged per stimulus to cut down background interference. Signal capture was completed with a Tucker-Davis Technologies system (Alachua, FL, USA). Auditory threshold values at every test frequency were judged by the clear emergence of wave II on recorded ABR waveforms. DPOAE signals were also catch by using the Tucker-Davis Technologies system. Primary tone pairs f1 and f2 were set at a fixed frequency ratio of 1.22, with f2 stimulus intensity reduced by 5 dB relative to f1; each test frequency corresponded to the geometric mean of the two primary tones. Roughly 200 repeated waveform traces were averaged at each frequency point to generate steady 2f1−f2 distortion product signals, and DPOAE signal amplitudes were quantified from these stable recordings.

### 2.4. Immunofluorescence Staining

Inner ear immunofluorescence staining was performed using standard laboratory protocols [20]. Primary antibodies including anti-MTF1 (ab183897, Abcam, Cambridge, UK, 1:200), anti-CtBP2 (612044, BD Biosciences, Franklin Lakes, NJ, USA, 1:200), and anti-myosin7a (256790, Proteus Bio-Sciences, Ramona, CA, USA, 1:500) were employed for tissue labeling at fixed dilutions. Mice were euthanized by cervical dislocation, and isolated inner ear tissues were fixed overnight in 4% paraformaldehyde at 4 °C. Specimens were decalcified in 10% EDTA for 5–6 h at room temperature, rinsed thoroughly with PBS, and microdissected under a stereomicroscope. Tissues were permeabilized with 0.3% Triton X-100 for 15 min at 25 °C, followed by 1 h of blocking with containing 5% bovine serum albumin (BSA). Tissues were incubated with diluted primary antibodies overnight at 4 °C. After repeated PBS washing, corresponding secondary antibodies were applied for 1 h at room temperature. Following further PBS rinses, samples were counterstained and mounted with DAPI (D9542, Sigma-Aldrich, St. Louis, MO, USA). Fluorescent images were acquired via a super-resolution laser scanning confocal microscope (Olympus FV1000, Olympus, Tokyo, Japan) for subsequent histological analysis.

### 2.5. Western Blot

Mouse cochlear tissues were homogenized in RIPA lysis buffer containing 1% PMSF, followed by 20 min of ice-cold incubation. Samples were heated at 100 °C for 10 min and centrifuged at 13,000× *g* for 5 min at 4 °C. The resulting supernatants were harvested, and total protein concentrations were quantified using a micro-BCA protein assay. An equal quantity of protein was separated on 10–12% SDS-PAGE gels via electrophoresis and subsequently transferred onto polyvinylidene fluoride membrane. Membranes were blocked with 5% skim milk to suppress non-specific binding, then incubated with primary antibodies overnight at 4 °C. The primary antibody includes anti-MTF1 (ab183897, Abcam, 1:1000), anti-TBK1 (83686-3-RR, proteintech, Wuhan, China, 1:1000), anti-STING (19851-1-AP, proteintech, Wuhan, China, 1:1000), anti-STING (19851-1-AP, proteintech, Wuhan, China, 1:1000), anti-cGAS (26416-1-AP, proteintech, Wuhan, China, 1:1000). Following thorough rinsing with TBST, HRP-conjugated secondary antibodies were applied for 2 h at 25 °C. Target protein bands were visualized using enhanced chemiluminescence reagents and scanned with a ChemiDoc XRS system (Hercules, CA, USA). β-Actin was used as an internal reference to verify consistent protein loading across all experimental groups. Relative band intensity was quantified semi-quantitatively via ImageJ (Version 1.52, NIH, Bethesda, MD, USA).

### 2.6. Real-Time Quantitative PCR

Total RNA was isolated from mouse cochlear specimens with TRIzol reagent (15596026, Thermo Fisher Scientific, Waltham, MA, USA) following standard phase separation procedures. Tissue homogenates were incubated at ambient temperature for 10 min before chloroform addition, and phase separation was achieved through subsequent centrifugation for 15 min. The upper aqueous RNA-containing fraction was collected, and RNA precipitation was induced with isopropanol. Recovered RNA pellets underwent two rounds of washing with 75% ethanol, air-dried, and resuspended in RNase-free water. Reverse transcription for cDNA generation was performed using the PrimeScript RT Reagent Kit (Takara, Kusatsu, Japan) in strict accordance with the manufacturer’s specifications. Quantitative real-time PCR was run on a StepOnePlus Real-Time PCR System (Applied Biosystems, Foster City, CA, USA) with SYBR Premix Ex Taq (Takara) and custom gene-specific primers. Relative gene expression values were computed using the 2^−ΔΔCt^ algorithm, and GAPDH was selected as the housekeeping gene for data normalization.

### 2.7. RNA Sequencing

Transcriptomic profiling via RNA sequencing was adopted to identify transcriptional variations between young and old mice. Cochlear specimens were dissected first, from which total RNA was purified to construct sequencing libraries. Library construction encompassed RNA fragmentation, sequencing adapter linkage and target fragment amplification. The Illumina NovaSeq6000 (San Diego, CA, USA) workstation was utilized to generate raw sequencing reads. Bioinformatic pipelines were applied to screen differentially expressed genes and execute functional pathway enrichment. Highly enriched gene clusters were further manually annotated to verify precise functional categorization and pathway attribution. Three independent biological replicates were established for each experimental group, and each biological replicate consisted of combined bilateral cochlear tissues harvested from a single mouse to guarantee adequate sample homogeneity. Differentially expressed genes (DEGs) were screened according to unified criteria: absolute fold change (|FC|) ≥ 1.2 and false discovery rate (FDR)-adjusted *p*-value < 0.05.

### 2.8. Cell Culture Conditions and Cell Viability

All cellular assays were performed following standardized culture workflows described in our prior reports. HEI-OC1 cells were maintained in high-glucose DMEM supplemented with 10% fetal bovine serum (Cat. 11054001; Gibco, Thermo Fisher Scientific, Waltham, MA, USA). Cultures were incubated for 24 h at 37 °C within a humidified incubator supplied with 5% CO_2_. Cellular density and viability were examined under light microscopy to verify satisfactory growth status. Only cells at exponential proliferation, ranging from 10^5^ to 10^6^ cells/mL, were retained for subsequent experimental interventions. Cell viability was measured via the Cell Counting Kit-8 (CCK-8, Beyotime Institute of Biotechnology, Nantong, China). Cell pellets were rinsed with phosphate-buffered saline (PBS), and all manipulations were performed in line with the kit’s official guidelines. Following brief incubation at ambient temperature, viable cell populations were visualized.

### 2.9. Zinc Supplementation and Depletion (TPEN) Treatments

Excess intracellular zinc causes cytotoxicity relative to physiological zinc levels. Based on published studies and pilot tests, an in vitro zinc-overload model was established by culturing HEI-OC1 auditory cells with serially diluted zinc glycinate (0, 5, 10, 20, 50 μM) in serum-free medium for 4 h. For zinc-deficient cell models, cells were treated with gradient doses of the zinc chelator TPEN (0–20 µM; Sigma, St. Louis, MO, USA) under identical serum-free conditions over 4 h. BSA and DMSO were used as vehicle controls for zinc-replete and zinc-depleted groups, respectively. All treatments were repeated in three independent biological experiments.

### 2.10. Cell Transfection

HEI-OC1 cells were transfected with an overexpression plasmid to overexpress MTF-1 gene. Empty vector was purchased from Thermo Fisher scientific Company (Waltham, MA, USA). cDNAs that were used for the construction of overexpression plasmid of MTF-1 were synthesized and constructed into the vector. The corresponding RNA sequences were obtained from the National Center for Biotechnology Information (NCBI) database. Empty vector was used as a negative control. HEI-OC1 cells were seeded in 6-well plates and cultured to 80% confluence. The samples were transfected with 1 µg of plasmid in a turbofect transfection reagent (R0531, Thermo Scientific, Waltham, MA, USA) for 24 h according to the manufacturer’s protocols. The transfection efficiencies were validated by RT-qPCR.

### 2.11. Transmission Electron Microscopy

Inner ear tissues collected from cervically dislocated mice were processed for transmission electron microscopy (TEM). Dissected samples underwent overnight primary fixation in 2.5% glutaraldehyde (G5882, Sigma-Aldrich, St. Louis, MO, USA) at 4 °C prior to decalcification in 10% EDTA buffer. After complete washing steps, isolated organs of Corti received secondary fixation in 1% osmium tetroxide (05500, Sigma-Aldrich, St. Louis, MO, USA) over a 2 h period to stabilize subcellular ultrastructures. Samples were sequentially dehydrated using graded ethanol solutions and washed with acetone before embedding in fresh epoxy resin. Ultrathin slices measuring 60–90 nm were generated and placed onto copper grids. Ultrastructural photographs were captured with transmission electron microscopes (FEI Tecnai G220 TWIN, Hillsboro, OR, USA).

### 2.12. Mitochondrial Membrane Potential Detection

Mitochondrial membrane potential (MMP) and mitochondrial functional status in HEI-OC1 cells were assessed using JC-1 (C2003S, Beyotime, Nantong, China) and TMRE (C2001S, Beyotime, Nantong, China) staining kits, respectively. All staining operations were performed fully compliant with the manufacturer’s standard protocols. Following experimental treatment, cell samples were collected and incubated according to the official reagent instructions. Fluorescence signals were visualized and recorded using a fluorescence microscope (Leica DMI 3000 B, Leica, Wetzlar, Germany). Acquired imaging data were further analyzed and merged with ImageJ software (Version 1.52, NIH, Bethesda, MD, USA) for subsequent quantitative evaluation.

### 2.13. Statistical Analysis

All quantitative data are presented as the mean ± standard deviation (SD), with all measurements derived from no fewer than three independent biological replicates, each containing three technical repetitions. Statistical processing of experimental data was completed using GraphPad Prism 8.0 (GraphPad Software, Version 8.0, Boston, MA, USA). Prior to statistical evaluation, all datasets were pre-screened for normal distribution and homogeneity of variance across experimental groups. Two-group statistical comparisons were conducted using a two-tailed unpaired Student’s *t*-test. For multi-group comparison scenarios, one-way ANOVA was adopted when the prerequisites of normality and uniform variance were satisfied. Upon a significant main effect of ANOVA, Tukey’s honestly significant difference (Tukey’s HSD) post hoc test was adopted for pairwise multiple comparisons to correct type I error. For the longitudinal ABR and DPOAE threshold data with repeated measurements on the same animals, we employed repeated-measures two-way analysis of variance (repeated-measures two-way ANOVA), with treatment group as the between-subjects factor and time as the within-subjects factor. When a significant overall effect was detected by repeated-measures ANOVA, Tukey’s HSD post hoc test was used for pairwise multiple comparisons between groups at each time point, as well as for pairwise comparisons across time points within each group. Regardless of the statistical methods applied, a *p*-value lower than 0.05 was regarded as the cutoff for significant statistical differences.

## 3. Results

### 3.1. Zinc Deficiency Accelerates the Onset of ARHL in C57BL/6J Mice

The inbred C57BL/6J strain serves as a classic and extensively investigated spontaneous model of ARHL. It is characterized by progressive high-frequency hearing loss emerging from early adulthood, which is highly consistent with the clinical manifestations of human presbycusis. Accumulated studies have verified that the period from 6 to 9 months of age corresponds to the early progressive stage of ARHL in C57BL/6J mice [21,22,23,24]. During this window, prominent elevations in the thresholds of ABR and DPOAE at high frequencies can be detected, outer hair cell lesions initiate at the basal turn of the cochlea, accompanied by mild mitochondrial damage and gradual synaptic pathological alterations [25]. These pathological alterations closely recapitulate the early-to-middle phase of human age-related hearing impairment, rendering C57BL/6J mice an ideal ARHL animal model. C57BL/6J mice were fed a zinc-deficient diet to establish a zinc deficiency model, while control mice were maintained on a normal diet. Auditory function was dynamically assessed in different treatment groups (Figure 1A). Serum zinc level measurements showed a significant decrease in the zinc-deficient group, with serum zinc concentrations progressively declining over time (Figure 1B). Similarly, cochlear zinc levels in the zinc-deficient group were significantly lower than those in the control group (Figure 1C). ABR results showed that at 2 months of age, at the beginning of the modeling period, the mice exhibited normal hearing. At 6 months of age, mice in the zinc-deficient group displayed significant high-frequency hearing loss (24, 32, and 40 kHz), which progressively worsened over time. At 8 and 10 months of age, auditory thresholds across all tested frequencies in the zinc-deficient group were significantly higher than those in the control group (Figure 1D). Furthermore, we evaluated hair cell survival using immunofluorescence staining of cochlear basilar membranes. Consistent with the ABR results, at 2 months of age, the three rows of outer hair cells exhibited a continuous arrangement without any loss. In the zinc-deficient group, outer hair cell loss was observed at the basal turn of the basilar membrane starting from 6 months of age. This hair cell loss progressively worsened over time and spread toward the middle and apical turns. At 8 and 10 months of age, the basilar membrane of the zinc-deficient group showed extensive outer hair cell loss (Figure 1E). Quantitative analysis of hair cells along the entire length of the basilar membrane further demonstrated that the rate of hair cell loss in the zinc-deficient group was significantly higher than that in the control group (Figure 1F). Collectively, our results indicate that long-term zinc deficiency leads to disruption of cochlear zinc homeostasis and damage to cochlear hair cells, thereby accelerating the onset of sensorineural hearing loss.

### 3.2. Zinc Glycinate Supplementation Significantly Ameliorates Age-Related Hearing Loss in C57BL/6J Mice

Having established that zinc homeostasis imbalance in the inner ear accelerates hearing loss, we further investigated the effects of maintaining zinc homeostasis on ARHL progression. C57BL/6J mice were chronically supplemented with zinc glycinate, and auditory function was dynamically assessed (Figure 2A). Serum and cochlear zinc levels were measured in the zinc-supplemented group. Both serum and cochlear zinc concentrations were significantly higher in the supplemented group than in the control group (Figure 2B,C). As shown in Figure 2D, auditory thresholds at different frequencies were compared between groups. Untreated mice exhibited progressive hearing loss with age, whereas zinc-supplemented mice showed significantly lower auditory thresholds across all tested frequencies, indicating that zinc supplementation partially improved auditory function (Figure 2D). To further analyze the effect of zinc glycinate on individual hearing loss, we plotted the threshold shifts in control and zinc-sufficient mice. Notably, zinc glycinate exhibited more pronounced ameliorative effects on hearing loss in C57BL/6J mice in the high-frequency range (Figure 2E). We next examined hair cell survival in the zinc-supplemented group. Consistent with the ABR findings, mice treated with zinc glycinate exhibited significantly reduced outer hair cell loss. As shown in Figure 2F, at 6 months of age, the control group showed sporadic outer hair cell loss, while the zinc glycinate-treated group displayed no hair cell loss. Similarly, at 8 and 10 months of age, outer hair cell loss was reduced in the zinc glycinate-treated group compared with the control group (Figure 2F). Furthermore, we assessed the activities of antioxidant enzymes in the cochleae of zinc-supplemented mice. The results showed that zinc supplementation significantly increased the activities of SOD, GSH, and GPX4, suggesting that zinc glycinate enhances cochlear antioxidant capacity, thereby reducing aging-driven oxidative damage to hair cells (Figure 2G–I). Collectively, these results further confirm that maintaining zinc homeostasis in the inner ear is beneficial for mitigating ARHL in C57BL/6J mice.

### 3.3. Zinc Glycinate Supplementation Significantly Ameliorates Synaptic Damage and Restores Cochlear Amplification Function in C57BL/6J Mice

Cochlear synaptopathy is an early and critical pathological event in ARHL, occurring prior to hair cell death and leading to hidden hearing loss [26]. Age-related synaptic damage is a complex pathological process mediated by the synergistic action of multiple factors and signaling pathways, with oxidative damage serving as one of its core mechanisms. With advancing age, the activities of key antioxidant enzymes in hair cells gradually decline, resulting in excessive accumulation of intracellular ROS. ROS can directly disrupt key synaptic proteins or activate oxidative stress-sensitive signaling pathways, ultimately leading to synaptic dysfunction. To further determine whether zinc glycinate protects against age-related synaptic damage, C57BL/6J mice were orally administered zinc glycinate daily from 2 months of age, and the protective effect on cochlear amplification was dynamically evaluated (Figure 3A). As shown in Figure 3B, mice treated with zinc glycinate exhibited significantly elevated DPOAE amplitudes compared with untreated mice. With increasing age, DPOAE amplitudes in C57BL/6J mice showed a declining trend, which was significantly reversed by zinc glycinate supplementation (Figure 3B). Quantitative analysis of DPOAE output values at different frequencies further revealed that at 10 months of age, zinc glycinate-treated mice displayed significantly enhanced DPOAE outputs at all tested frequencies, indicating that zinc glycinate more effectively protects outer hair cell function and improves cochlear amplification in aged mice (Figure 3C). To further assess the protective effect of zinc glycinate on cochlear synaptic structures, immunofluorescence staining was performed to visualize afferent synapses in the inner ear of mice from different treatment groups. As shown in Figure 3D, at 10 months of age, compared with control mice, the zinc glycinate-treated group exhibited a significant increase in the number of afferent synapses in inner hair cells (Figure 3D). Specifically, the numbers of afferent synapses in the apical, middle, and basal turns of the control group were 9.67 ± 1.21, 8.83 ± 1.17, and 7.50 ± 1.05, respectively, while those in the zinc glycinate-treated group were 14.17 ± 1.47, 12.50 ± 1.05, and 12.83 ± 1.33 (Figure 3E). Our findings indicate that zinc glycinate supplementation significantly alleviates synaptic oxidative damage in the inner ear of aged C57BL/6J mice, suggesting its capacity to preserve synaptic connections and mitigate neurodegeneration.

### 3.4. Zinc Glycinate Suppresses the cGAS-STING Pathway by Upregulating MTF-1

To further explore the potential molecular mechanisms underlying the protective effects of zinc glycinate against hair cell damage, transcriptome sequencing analysis was performed on cochleae from aged mice (Figure 4A). Differential gene expression analysis revealed that, compared with young mice, 559 genes were significantly upregulated and 437 genes were significantly downregulated in the cochleae of aged mice (Figure 4B). Subsequent Kyoto Encyclopedia of Genes and Genomes (KEGG) pathway enrichment analysis showed significant enrichment of pathways related to energy metabolism and immune responses, including the MAPK and cGAS-STING signaling pathways (Figure 4C). As shown in Figure 4D, the heatmap displays the top 20 genes with the most significant expression differences, among which the expression of the zinc finger transcription factor MTF-1 was markedly downregulated (Figure 4D). MTF-1 is a crucial zinc-finger protein that functions as a master regulator of cellular stress responses, maintaining metal ion homeostasis and counteracting oxidative stress and other stressors by modulating the expression of specific genes. Therefore, the downregulation of MTF-1 may be a key molecular driver of ARHL progression. As shown in Figure 4E, quantitative real-time PCR further confirmed that MTF-1 expression in the cochleae of aged mice was significantly downregulated, while zinc glycinate treatment significantly reversed this downregulation (Figure 4E). Furthermore, Western blot results demonstrated that zinc glycinate significantly restored the age-related decline in MTF1 expression in the cochlea at the protein level (Figure 4F,G). In addition, we found activation of the cGAS-STING signaling pathway in the cochleae of aged mice, with significant upregulation of TBK1, MB21D1, and TMEM173 expression. Zinc glycinate treatment significantly reversed these upregulations (Figure 4H). As shown in Figure 4I, compared with young mice, aged mice exhibited significantly elevated expression of TBK1, cGAS, and STING, which was suppressed by zinc glycinate treatment, suggesting that zinc glycinate inhibits cGAS-STING pathway activation. Intracellular ROS accumulation can activate the cGAS-STING pathway, which further suppresses the antioxidant system and drives persistent inflammation and cell damage. Therefore, zinc glycinate may maintain antioxidant system stability by upregulating MTF-1 expression, thereby inhibiting cGAS-STING pathway activation.

### 3.5. Zinc Deprivation Leads to Mitochondrial Dysfunction via Downregulation of MTF-1

To further explore the potential molecular mechanisms underlying zinc homeostasis dysregulation-induced cochlear cell damage, we first established a zinc-deprivation model in HEI-OC1 cells using the zinc ion chelator TPEN (Figure 5A). CCK-8 assay was performed to evaluate cell viability after treatment with different concentrations of TPEN. The results showed that cell viability significantly decreased when the TPEN concentration reached 10 μM (Figure 5B). Therefore, a TPEN concentration of 5 μM, which had no significant effect on cell viability, was selected for subsequent experiments. We next evaluated the changes in MTF-1 expression following TPEN treatment. The results showed that TPEN treatment led to a decrease in MTF-1 expression (Figure 5C). Quantitative analysis revealed that MTF-1 protein expression in the TPEN-treated group was reduced by 75.3 ± 4.37% compared with the control group (Figure 5D). Similarly, quantitative analysis of immunofluorescence intensity further confirmed a significant decrease in intracellular MTF-1 expression. Specifically, MTF-1 expression in the TPEN-treated group was decreased by 58.28 ± 5.76% relative to the control group (Figure 5E). β-Galactosidase staining was performed to assess cellular senescence. Compared with the control group, TPEN treatment significantly increased the number of β-galactosidase-positive senescent cells, indicating that zinc deprivation induces cellular senescence (Figure 5F). Furthermore, we evaluated the effect of zinc homeostasis dysregulation on the cGAS-STING pathway. PCR results revealed that mRNA levels of cGAS, STING, and TBK1 were significantly elevated in the TPEN-treated group, suggesting activation of the cGAS-STING pathway (Figure 5G). As shown in Figure 5H, Western blot analysis also confirmed a significant increase in the protein expression of cGAS, STING, and TBK1. In addition, we assessed the impact of TPEN treatment on mitochondrial function using JC-1 and TMRM probes. For the JC-1 fluorescent probe, normal mitochondria exhibit red fluorescence, indicating high ΔΨm, while damaged mitochondria display green fluorescence. As shown in Figure 5I, the TPEN-treated group showed markedly reduced red JC-1 fluorescence and increased green fluorescence, reflecting a decrease in mitochondrial ΔΨm (Figure 5I). For TMRM staining, fluorescence intensity is positively correlated with ΔΨm. Quantitative analysis of immunofluorescence showed that TPEN treatment significantly reduced red fluorescence intensity (Figure 5J,K). Collectively, these data indicate that intracellular zinc homeostasis imbalance leads to mitochondrial dysfunction, which may be associated with downregulation of MTF-1 expression, thereby activating the cGAS-STING pathway.

### 3.6. Zinc Glycinate Protects Against D-Gal Induced Cellular Damage by Upregulating MTF-1

To further clarify the protective effect of zinc glycinate against cellular senescence and its underlying molecular mechanisms, we established a D-gal induced cellular senescence model (Figure 6A). Cell viability was assessed after treatment with different concentrations of D-gal. The results showed that cell viability significantly decreased when the D-gal concentration reached 10 mg/mL (Figure 6B). Therefore, a D-galactose concentration of 10 mg/mL was used in subsequent experiments. We found that zinc glycinate treatment significantly reversed the D-gal-induced decrease in cell viability (Figure 6C). We further investigated the molecular mechanisms by which zinc glycinate counteracts D-gal-induced cellular damage. PCR results showed that D-gal decreased MTF-1 mRNA levels, while zinc glycinate treatment reversed this effect (Figure 6D). Western blot and immunofluorescence staining were further performed to examine MTF-1 protein expression. The results demonstrated that D-gal treatment significantly reduced MTF-1 expression, whereas zinc glycinate largely restored its expression, suggesting that zinc glycinate may exert its protective effect through upregulation of MTF-1 (Figure 6E). Immunofluorescence staining further confirmed that zinc glycinate reversed the D-gal induced downregulation of MTF-1 expression (Figure 6F). In addition, transmission electron microscopy was used to observe ultrastructural changes in the cells. The results showed that D-gal treatment caused disruption of mitochondrial structure, characterized by vacuolization and loss of cristae, whereas zinc glycinate treatment significantly reversed these pathological changes, with mitochondria exhibiting a nearly normal morphology (Figure 6G). Functional assessments further demonstrated that D-gal treatment decreased mitochondrial membrane potential, while zinc glycinate treatment significantly restored mitochondrial membrane potential, indicating marked improvement in mitochondrial function (Figure 6H). Furthermore, we evaluated the expression changes in cGAS, STING, and TBK1. The results showed that D-galactose treatment significantly increased the expression of TBK1, cGAS, and STING, suggesting activation of the cGAS-STING signaling pathway. Compared with the D-gal group, the zinc glycinate-treated group exhibited significantly decreased expression of cGAS, STING, and TBK1, indicating that zinc glycinate treatment significantly inhibited cGAS-STING pathway activation (Figure 6I). In summary, these data indicate that the protection conferred by zinc glycinate against D-gal induced damage may be mediated, at least in part, through upregulation of MTF1 and inhibition of the cGAS-STING pathway.

### 3.7. Overexpression of MTF-1 Significantly Ameliorates D-Gal Induced Cellular Damage

To further determine whether MTF-1 is a key molecule mediating the anti-aging effects of zinc glycinate, we first established an MTF-1 overexpression cell model (Figure 7A). Validation of MTF-1 expression in the overexpressing cells showed that MTF-1 mRNA levels were increased by approximately 2.14-fold (Figure 7B). Western blot analysis further confirmed that MTF-1 protein expression was significantly elevated in the MTF-1 overexpression group, demonstrating successful construction of the MTF-1 overexpression cell model (Figure 7C,D). To further elucidate the role of MTF-1 in D-galactose-induced cellular damage, we examined apoptosis in different treatment groups. As shown in Figure 7E, D-galactose induced apoptosis, whereas MTF-1 overexpression significantly reduced D-gal induced apoptosis (Figure 7E). We also examined the regulatory effect of MTF-1 overexpression on the cGAS-STING signaling pathway. Western blot results showed that D-galactose upregulated the expression of TBK1, cGAS, and STING, whereas endogenous overexpression of MTF-1 significantly suppressed the expression of these proteins, suggesting that MTF-1 inhibits cGAS-STING pathway activation (Figure 7F). Furthermore, we evaluated mitochondrial function. As shown in Figure 7G, strong red fluorescence (JC-1 aggregates) was observed in the control and MTF-1 overexpression groups, while D-galactose treatment resulted in decreased JC-1 aggregate fluorescence and increased JC-1 monomer fluorescence. Endogenous MTF-1 overexpression effectively reversed these changes and ameliorated D-gal induced mitochondrial dysfunction (Figure 7G). These preliminary results indicate that downregulation of MTF1 is involved in the pathological process of D-gal-induced cellular damage, and similarly, upregulation of MTF1 significantly alleviates D-gal-induced cellular injury. Considering that zinc glycinate treatment partially preserves MTF-1 expression against D-gal induced downregulation, we speculate that MTF-1 may be one of the key molecules mediating the auditory protective effect of zinc glycinate.

## 4. Discussion

ARHL is the most prevalent chronic sensorineural degenerative disorder among the elderly population worldwide, severely impairing daily communication, social participation, mental health, and cognitive function [27,28]. Current clinical interventions, such as hearing aids and cochlear implants, can only compensate for auditory function and cannot reverse endogenous cochlear degeneration [29]. Moreover, no disease-modifying pharmacological therapy is yet available to halt the progression of cochlear aging. Excessive oxidative stress and mitochondrial dysfunction are widely recognized as major drivers of ARHL, leading to degeneration of hair cells, synapses, and spiral ganglion neurons [30]. Cochlear hair cells, which serve as the primary sensory receptors that perceive sound wave vibrations and convert them into electrical signals, undergo functional decline, morphological atrophy, and numerical reduction with advancing age [26,31]. In adult mammals, loss of cochlear hair cells and spiral ganglion neurons is irreversible. Although some studies have achieved partial hair cell regeneration through genetic reprogramming, the newly generated hair cells still lack the functional maturity of native hair cells [32]. Therefore, understanding the mechanisms of cochlear hair cell damage and developing protective strategies are critical for the prevention and treatment of hearing loss. In recent years, advances in molecular biology have identified multiple pathways involved in ARHL pathogenesis, including oxidative stress, inflammatory responses, apoptosis, mitochondrial dysfunction, ferroptosis, autophagy, and genetic variations [33,34]. Furthermore, environmental factors and systemic comorbidities, such as long-term noise exposure, hypertension, diabetes, and hyperlipidemia, can accelerate ARHL progression [35,36]. Notably, nutritional deficiencies have gained increasing attention as important non-genetic risk factors, complementing existing research on the pathogenesis of ARHL [37]. Nutritional deficiencies may indirectly or directly contribute to the development and progression of ARHL by affecting key enzyme activities in the inner ear, regulating energy metabolism, and modulating auditory-related molecular pathways [38,39]. Studies have shown that deficiencies in vitamins, magnesium, iron, and zinc also increase the risk of hearing loss. The incidence of sudden sensorineural hearing loss in populations with magnesium deficiency is 2.3 times higher than in normal populations, and magnesium supplementation can improve speech discrimination scores by approximately 23% in ARHL patients while reducing the risk of noise-induced hearing loss [40]. The synergistic deficiency of vitamin D and calcium is also noteworthy, the prevalence of vitamin D deficiency among people over 50 years of age is as high as 60%, and individuals with insufficient vitamin D have a 40% higher risk of hearing loss than those with adequate levels [41]. Vitamin D insufficiency affects calcium absorption, jointly contributing to the aging of auditory function. Additionally, supplementation with vitamin C and vitamin E, together with other antioxidants, can protect auditory function by scavenging reactive oxygen species and reducing mitochondrial DNA damage [42,43]. Current evidence has clearly established a close association between nutritional deficiencies and the risk of ARHL. However, the molecular mechanisms by which nutritional deficiencies regulate auditory system aging remain to be further elucidated, which may provide new insights for early prevention and precision intervention in ARHL.

Zinc is one of the 14 essential trace elements for the human body and is the second most abundant trace element after iron, often referred to as the “element of life.” Zinc serves as a structural, catalytic, and signaling component in both intracellular and intercellular communication, participating in various biological processes, including growth and development, metabolism, immune regulation, and neural transmission [44,45]. Studies have shown that zinc is abundant in the cochlea and plays an important role in maintaining inner ear microenvironment homeostasis [46]. As a constituent and activity regulator of over 300 enzymes, zinc participates in the antioxidant defense system, thereby reducing oxidative damage to cochlear hair cells caused by reactive oxygen species [47,48]. In addition to its direct enzymatic regulatory functions, zinc also stabilizes the Nrf2 antioxidant axis, promoting transcriptional upregulation of multiple antioxidant proteins and further enhancing cellular redox capacity [49,50]. Superoxide dismutase, glutathione, and catalase constitute the major intracellular antioxidant defense system [51]. In zinc-deficient C57BL/6J mice, we observed earlier onset of high-frequency hearing loss, accompanied by significantly reduced levels of SOD, GSH, and GPX4 in the cochlea, as well as severe mitochondrial depolarization and cristae disruption. Conversely, zinc glycinate treatment effectively replenished cochlear SOD, GSH, and GPX4 levels, reduced oxidative damage to cochlear hair cells and synapses, and ameliorated ARHL in C57BL/6J mice. Beyond direct enzymatic regulation, zinc-dependent MTF1 also enhances cellular antioxidant capacity at the transcriptional level. As a zinc-sensitive transcription factor, MTF-1 translocates into the nucleus upon elevated zinc concentrations, thereby activating the transcription of antioxidant and metal-detoxifying genes driven by metal-responsive elements [52,53]. Our transcriptome sequencing results revealed that MTF-1 expression was significantly downregulated in aged cochlear tissues, a phenomenon that was completely reversed by zinc glycinate treatment. In vitro, TPEN-mediated zinc deprivation suppressed MTF-1 expression in HEI-OC1 cells, accompanied by mitochondrial dysfunction and exacerbated cellular senescence. Conversely, zinc glycinate treatment significantly reversed the D-gal induced downregulation of MTF-1 expression, indicating that MTF-1 is responsive to cellular zinc homeostasis imbalance. Furthermore, overexpression of MTF-1 independently rescued D-gal induced mitochondrial damage. Given that zinc glycinate restored the D-gal induced downregulation of MTF-1 expression, we hypothesize that MTF-1 may be a key molecule mediating the auditory protective effect of zinc glycinate. Adequate intracellular zinc ions maintain mitochondrial structural integrity by enhancing the antioxidant system and regulating MTF-1 expression, thereby protecting cochlear hair cells from age-related oxidative damage.

MTF-1 is a master transcriptional regulator of cellular zinc homeostasis and metal stress responses. Under physiological conditions with adequate zinc, zinc ions bind to the zinc-finger domains of MTF-1, promoting its nuclear translocation [54]. MTF-1 subsequently binds to metal-responsive elements (MREs), thereby driving the transcription of antioxidant genes and metal-detoxifying genes [55]. Oxidized mitochondrial DNA (mtDNA), upon leakage into the cytoplasm, binds to cGAS, generating cGAMP, which in turn activates STING and triggers TBK1 phosphorylation. This sequentially activates IRF3-mediated type I interferon production and NF-κB-dependent secretion of pro-inflammatory cytokines [56]. In this study, we identified a direct link between MTF-1 and the cGAS-STING innate immune pathway in the cochlea. KEGG enrichment analysis of the aged cochlear transcriptome revealed significant enrichment of the cGAS-STING signaling pathway, accompanied by downregulation of MTF-1 expression. Zinc glycinate supplementation inhibited the upregulation of cGAS, STING, and TBK1. notably, this inhibitory effect was largely dependent on MTF-1 upregulation. In in vitro experiments, both D-gal induced senescence-like models and TPEN-mediated zinc-deficient cell models significantly downregulated MTF-1 expression and concurrently activated the core cGAS-STING signaling pathway. Notably, ectopic overexpression of MTF-1 significantly attenuated D-gal-induced oxidative injury, while zinc glycinate sustained MTF-1 expression levels. Accordingly, it is plausible that zinc glycinate exerts its protective effects against age-related oxidative damage, at least in part, via MTF-1. Sustained cGAS-STING hyperactivation serves as a key amplifying factor in age-related cochlear damage. Downstream inflammatory mediators further stimulate NOX/iNOS to generate additional ROS, forming a self-amplifying positive feedback loop between oxidative stress and inflammation [57,58]. The pathological vicious cycle between zinc homeostasis dysregulation and cGAS-STING activation leads to multi-layer cochlear degeneration. We propose a complete pathological cascade of age-related cochlear damage: physiological aging reduces intestinal zinc absorption and impairs inner ear zinc transport, disrupting cochlear zinc homeostasis. Zinc deficiency suppresses MTF-1 expression, weakens the cellular antioxidant defense system, and leads to excessive ROS accumulation. Excess ROS disrupts mitochondrial membrane integrity, opens the mPTP, and releases oxidized mtDNA into the cytoplasm, where it activates the cGAS-STING pathway [59,60]. This triggers early cochlear synaptopathy, progressive loss of outer hair cells, and permanent elevation of sensorineural hearing thresholds at high frequencies. Our study demonstrates that zinc glycinate supplementation can partially reverse or delay this process, and that the auditory protection conferred by zinc glycinate may be associated with maintaining MTF-1 expression and suppressing the cGAS-STING inflammatory signaling pathway.

We must admit that this research still has some limitations. All in vivo phenotypic verification was conducted exclusively on C57BL/6J inbred mice, a classic strain with genetically programmed spontaneous high-frequency ARHL. While this strain is ideal for mechanistic exploration of age-related cochlear degeneration, it cannot fully represent heterogeneous aging hearing loss occurring in outbred mouse strains or mixed-genotype animals. Genetic background determines the onset speed and lesion location of ARHL; thus, conclusions derived from C57BL/6J cannot be directly generalized to all mouse models of hearing aging. The current research is entirely preclinical animal and cellular research, lacking clinical verification from human peripheral blood, cochlear tissue samples or clinical cohort observation. Although we have completed animal–human dose conversion based on body surface area, interspecies differences in zinc absorption, metabolic rate and inner ear pathological characteristics inevitably restrict direct clinical extrapolation of our results. At present, we can only provide theoretical and experimental preclinical evidence for nutritional intervention against ARHL. We merely explored chelated zinc glycinate, without parallel comparison with inorganic zinc or other organic zinc chelate. We cannot distinguish whether the advantages of zinc glycinate stem from glycine ligand-assisted absorption, zinc ion itself, or their synergistic effects, nor can we judge whether other zinc formulations exert equivalent otoprotective effects. Our safety observation only lasted for the intervention cycle of this experiment, and long-term chronic safety monitoring is lacking. Long-term continuous zinc supplementation carries potential risks such as copper competitive malabsorption, iron metabolism disturbance and mild liver burden, which have not been quantitatively detected with long-term dynamic indicators in this study. Longer-term observations are needed, along with regular monitoring of the stable levels of trace elements, liver and kidney functions, and hematological indicators, in order to systematically assess the long-term safety of chronic oral administration of zinc glycinate.

## 5. Conclusions

Clinically, ARHL is only managed via hearing aids or cochlear implants that merely compensate auditory function, with no effective dietary or pharmaceutical interventions capable of reversing the underlying cochlear degenerative lesions triggered by nutritional imbalance. Our data demonstrate that long-term oral administration of zinc glycinate significantly improves ARHL and cochlear hair cell damage in C57BL/6J mice. As an oral nutritional micronutrient with well-documented, excellent biosafety, zinc glycinate possesses superior translational advantages over synthetic small-molecule inhibitors for long-term preventive application in elderly populations. Furthermore, this study reveals that MTF-1-dependent activation of the cGAS-STING innate immune pathway is involved in the pathological process of auditory system aging, providing a novel potential target for delaying or ameliorating the progression of presbycusis. Collectively, our findings confirm that zinc glycinate supplementation represents a safe, cost-effective, and easily implementable nutritional strategy with potential clinical value for the prevention and adjunctive management of ARHL. Moreover, this study offers preliminary mechanistic insights into how dietary trace element metabolism regulates sensory organ aging, providing new perspectives for micronutrient-based nutritional interventions against age-related degenerative diseases.

## Figures and Tables

**Figure 1:**
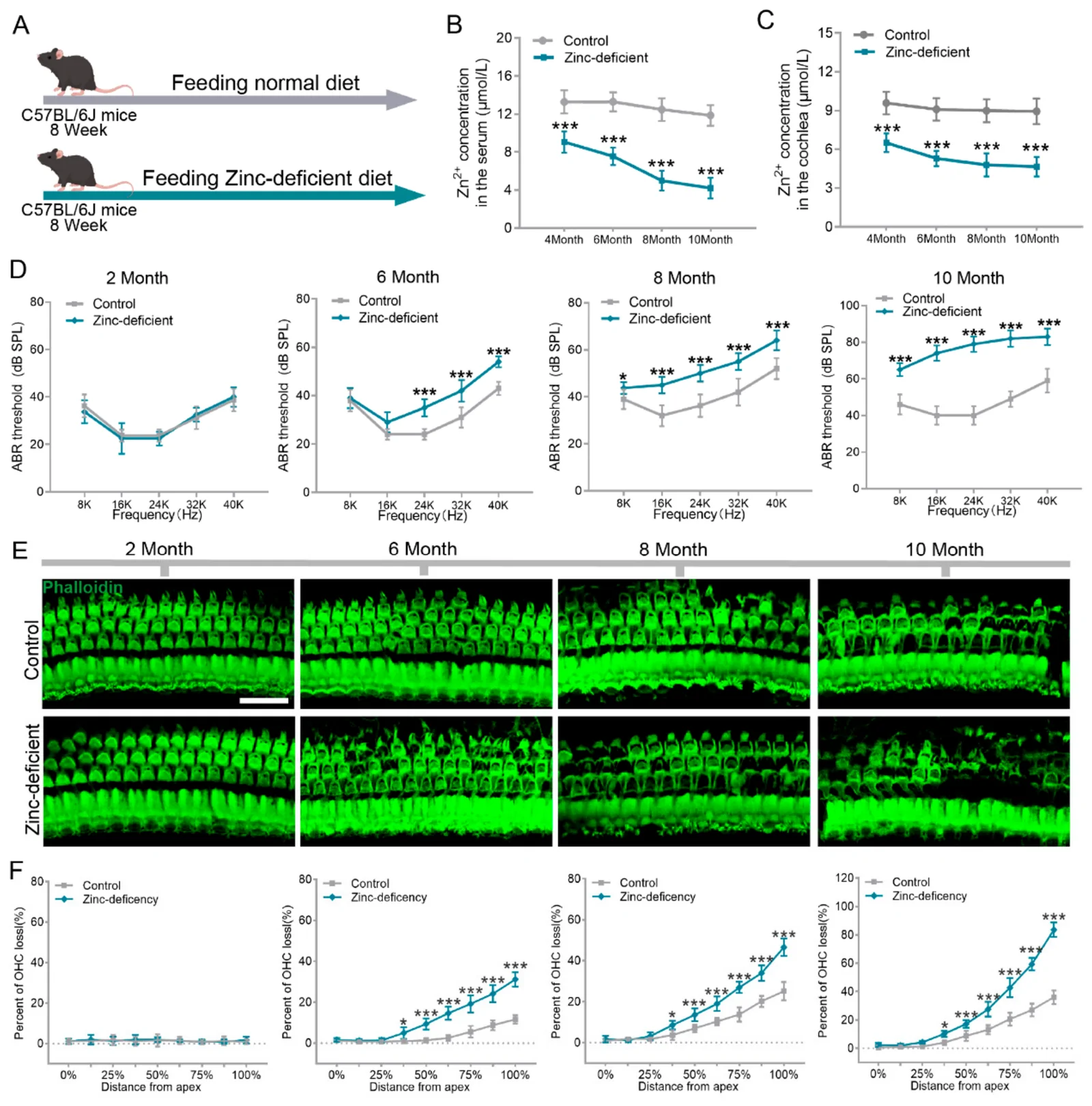
**Zinc deficiency accelerates the onset of ARHL in C57BL/6J mice.** (**A**) Establishment of a zinc-deficient mouse model. (**B**) Measurement of serum zinc levels in the zinc-deficient group (*n* = 5 in each group). (**C**) Measurement of cochlear zinc levels in the zinc-deficient group (*n* = 5 in each group). (**D**) Comparison of auditory function between the zinc-deficient group and the control group (*n* = 5 in each group). (**E**) Representative images of cochlear hair cell survival in the zinc-deficient group and the control group. (**F**) Quantitative analysis of the number of missing cochlear hair cells in different treatment groups (*n* = 5 in each group). * *p* < 0.05, *** *p* < 0.001. Scale bar in (**E**): 40 μm.

**Figure 2:**
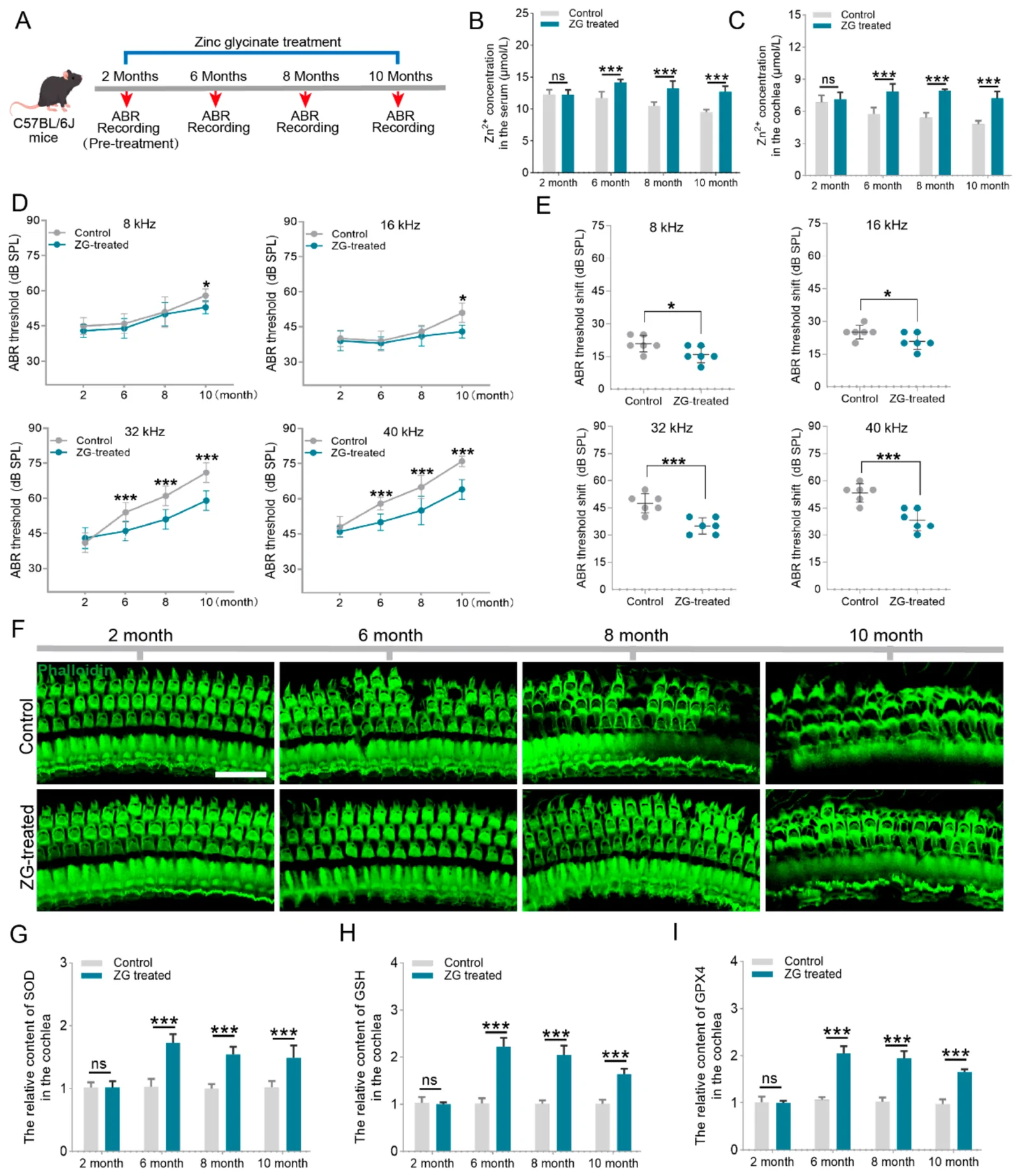
**Zinc glycinate supplementation significantly ameliorates age-related hearing loss in C57BL/6J mice.** (**A**) Outline for zinc glycinate treatment and ABR recordings in C57BL/6J mice. (**B**) Dynamic monitoring of serum zinc levels in mice (*n* = 5 in each group). (**C**) Dynamic monitoring of cochlear zinc levels in mice (*n* = 5 in each group). (**D**) Comparison of ABR thresholds between control and zinc glycinate-treated mice at 2, 6, 8, and 10 months of age (*n* = 5 in each group). (**E**) ABR threshold shifts in control and zinc glycinate-treated mice at 10 months of age (*n* = 5 in each group). (**F**) Representative images of cochlear hair cell survival in the zinc glycinate-treated group and the control group. (**G**–**I**) Dynamic detection of SOD1, GSH, and GPX4 levels in the cochlea (*n* = 5 in each group). * *p* < 0.05, *** *p* < 0.001, ns: no significance. Scale bar in (**F**): 40 μm.

**Figure 3:**
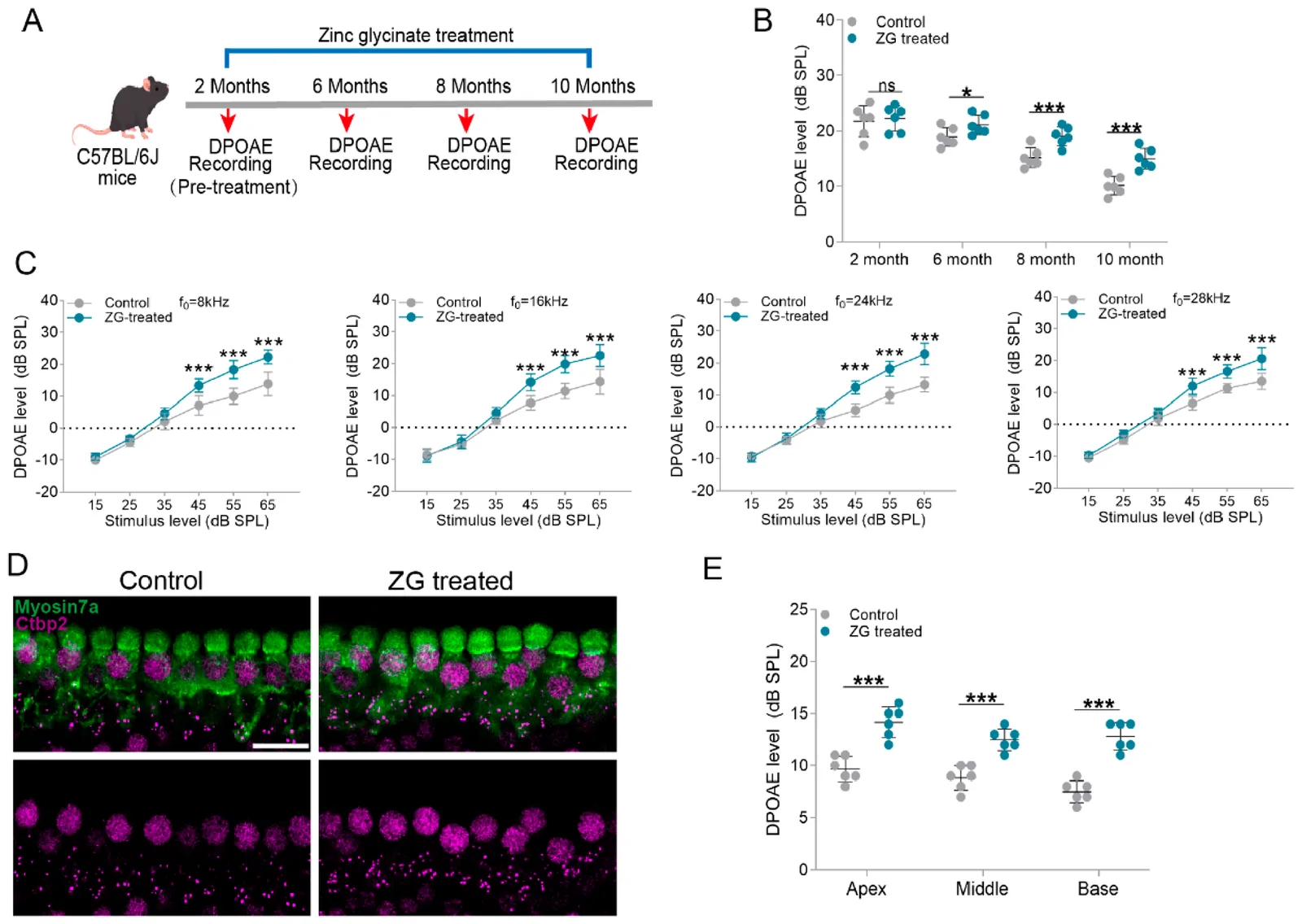
**Zinc glycinate supplementation significantly ameliorates synaptic damage in C57BL/6J mice.** (**A**) Outline for zinc glycinate treatment and DPOAE recordings in C57BL/6J mice. (**B**) Comparison of DPOAE output values between the control group and the zinc glycine group of mice (*n* = 5 in each group). (**C**) Input/output curves of DPOAE values from mice in different treatment groups at 10 months of age (*n* = 5 in each group). (**D**) CtBP2 immunofluorescence staining labeling inner hair cell synapses (*n* = 5 in each group). (**E**) Quantitative analysis of inner hair cell synapse numbers in different treatment groups (*n* = 5 in each group). * *p* < 0.05, *** *p* < 0.001, ns: no significance. Scale bar in (**D**): 40 μm.

**Figure 4:**
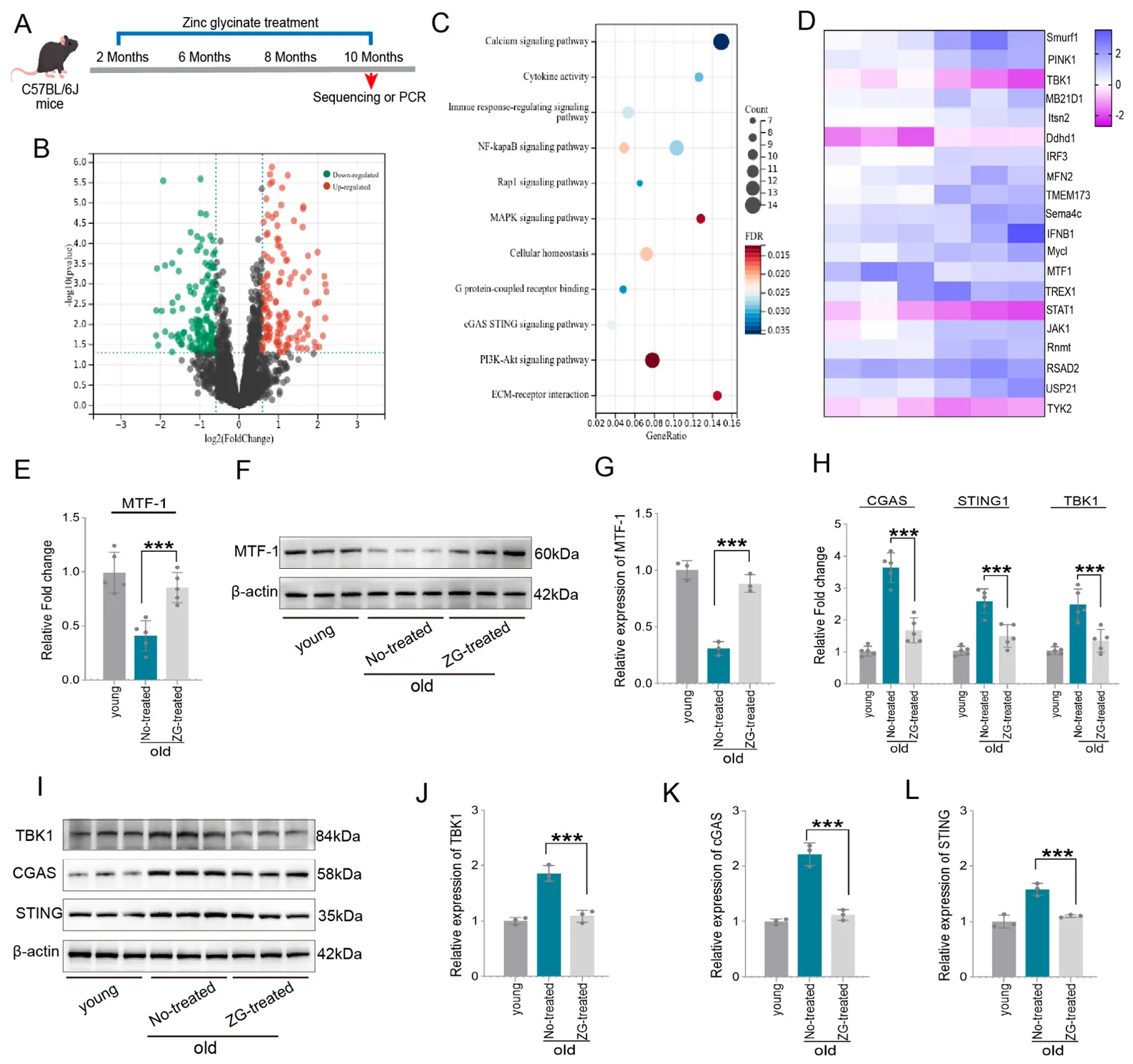
**Zinc glycinate suppresses the cGAS-STING pathway by upregulating MTF-1.** (**A**) Transcriptome sequencing analysis of cochleae from young mice (2 months old) and aged mice (10 months old). (**B**) Volcano plot showing differentially expressed genes (*n* = 3 in each group). (**C**) KEGG enrichment analysis of differentially expressed genes (*n* = 3 in each group). (**D**) Heatmap displaying the top 20 differentially expressed genes (*n* = 3 in each group). (**E**) qPCR analysis of MTF-1 mRNA levels in different groups (*n* = 5 in each group). (**F**) Western blot analysis of MTF-1 protein expression levels (*n* = 3 in each group). (**G**) Quantitative analysis of MTF-1 expression levels. (**H**) qPCR analysis of cGAS, STING, and TBK1 mRNA levels in different groups. (**I**) Western blot analysis of cGAS, STING, and TBK1 protein expression levels (*n* = 3 in each group). (**J**–**L**) Quantitative analysis of cGAS, STING, and TBK1 protein expression levels. *** *p* < 0.001.

**Figure 5:**
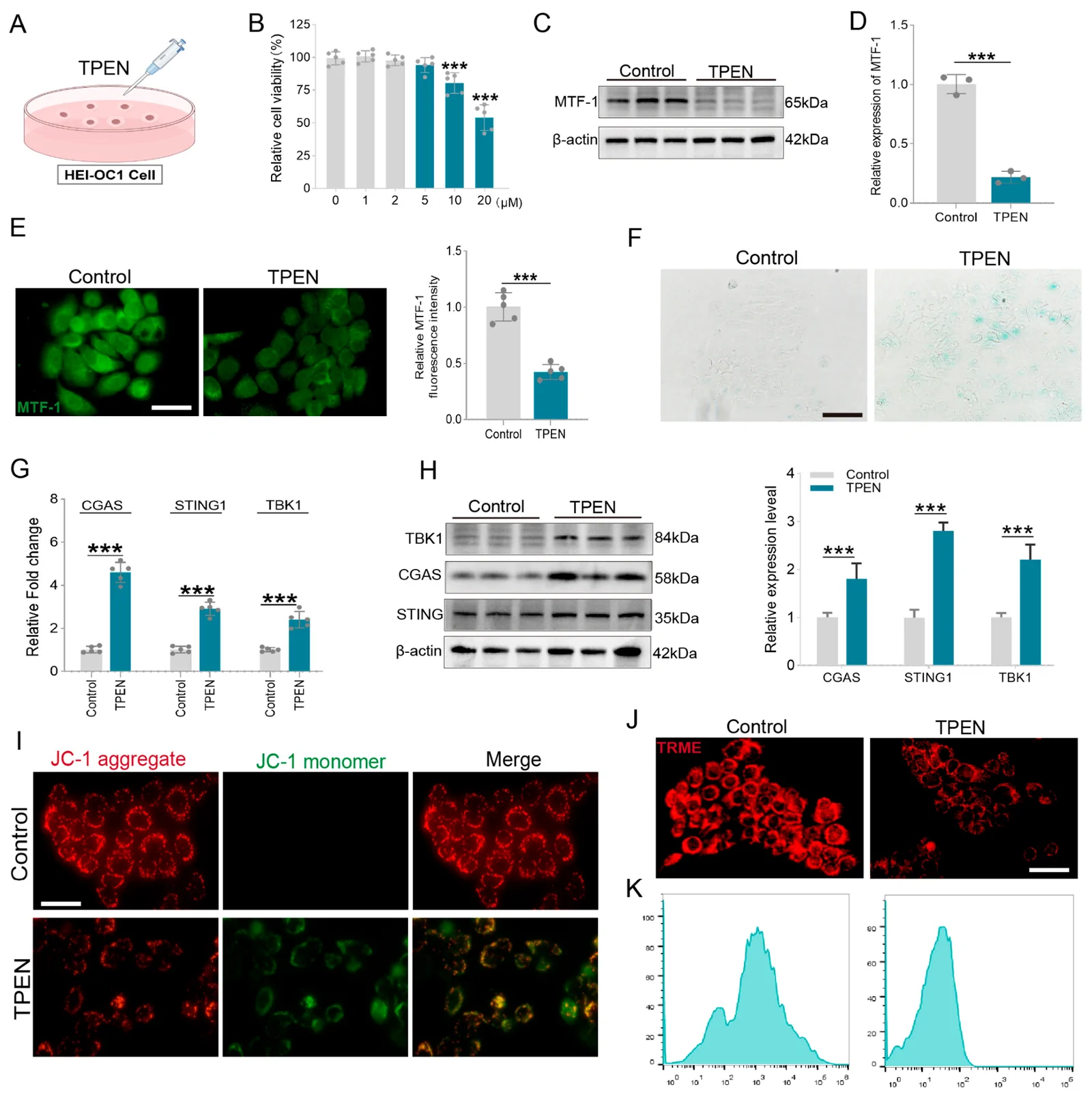
**Zinc deprivation leads to mitochondrial dysfunction via downregulation of MTF-1.** (**A**) TPEN treatment of HEI-OC1 cells to establish a zinc-deprivation cell model. (**B**) CCK-8 assay evaluating the effect of different concentrations of TPEN on cell viability (*n* = 5 in each group). (**C**) Western blot detection of MTF-1 expression changes (*n* = 3 in each group). (**D**) Quantitative analysis of MTF-1 expression changes. (**E**) Representative images of MTF-1 immunofluorescence (*n* = 5 in each group). (**F**) β-Galactosidase staining assessing cellular senescence in different treatment groups (*n* = 5 in each group). (**G**) qPCR detection of changes in cGAS, STING, and TBK1 mRNA levels (*n* = 5 in each group). (**H**) Western blot detection of changes in cGAS, STING, and TBK1 expression (*n* = 3 in each group). (**I**) Detection of mitochondrial membrane potential in different treatment groups using the JC-1 fluorescent probe (*n* = 5 in each group). (**J**) Detection of mitochondrial membrane potential using the TRME fluorescent probe (*n* = 5 in each group). (**K**) Quantitative analysis of TRME fluorescence intensity. *** *p* < 0.001. Scale bar 40 μm.

**Figure 6:**
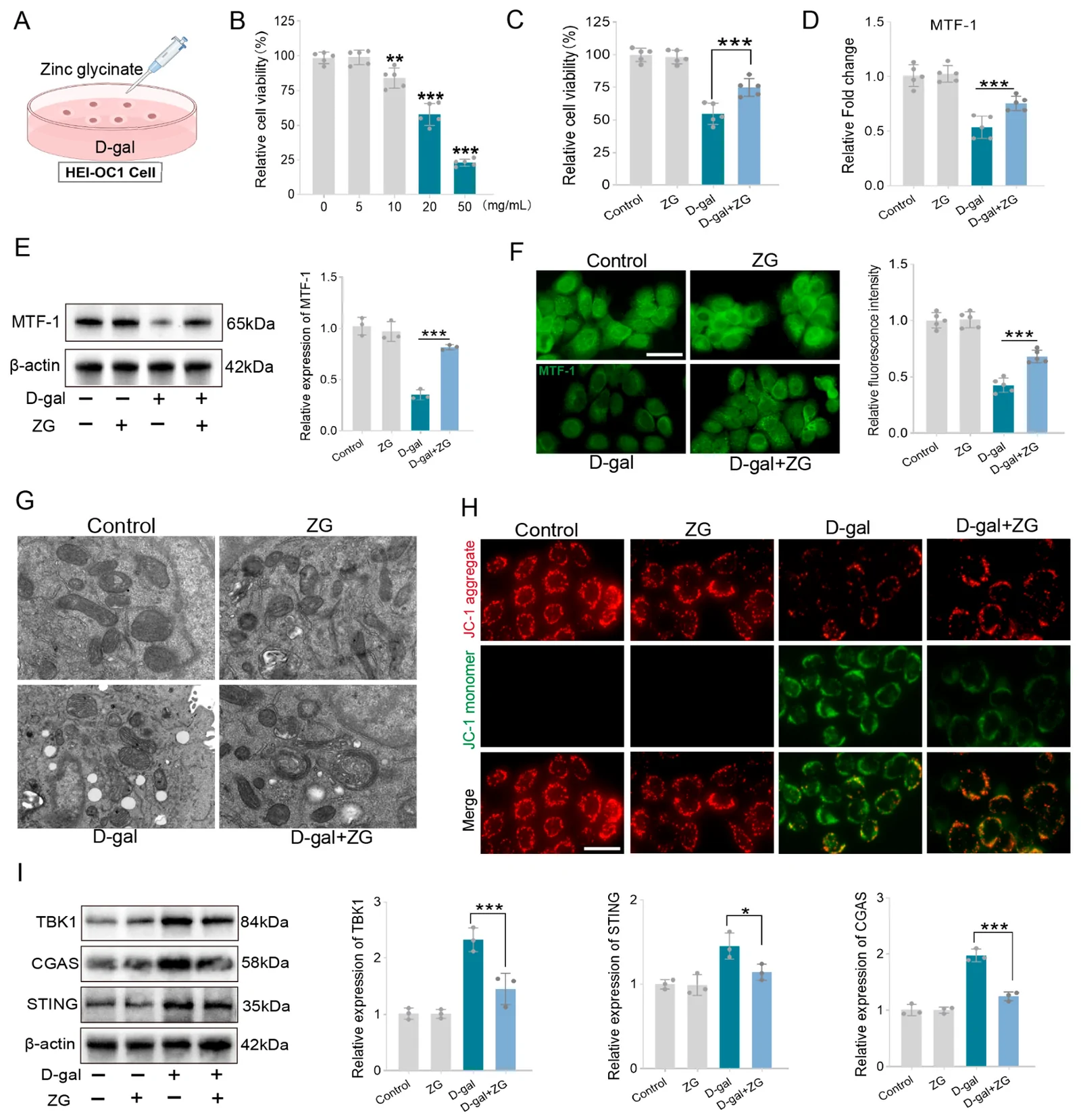
**Zinc glycinate protects against D-gal induced cellular damage by upregulating MTF-1.** (**A**) Establishment of a D-gal induced senescence-like cell model. (**B**) CCK-8 assay detecting cell viability in different concentrations of D-gal groups (*n* = 5 in each group). (**C**) Evaluation of the effect of zinc glycinate on D-galactose-induced cell viability (*n* = 5 in each group). (**D**) qPCR detection of MTF-1 mRNA levels in different treatment groups (*n* = 5 in each group). (**E**) Western blot analysis of MTF-1 expression changes in different treatment groups (*n* = 3 in each group). (**F**) Immunofluorescence staining evaluating MTF-1 expression changes in different treatment groups (*n* = 5 in each group). (**G**) Transmission electron microscopy detecting ultrastructural changes in different treatment groups (*n* = 3 in each group). (**H**) JC-1 fluorescent probe detecting mitochondrial membrane potential in different treatment groups (*n* = 5 in each group). (**I**) Western blot analysis of TBK1, cGAS, and STING expression changes in different treatment groups (*n* = 3 in each group). * *p* < 0.05, ** *p* < 0.01, *** *p* < 0.001. Scale bar (**F**,**H**) 40 μm.

**Figure 7:**
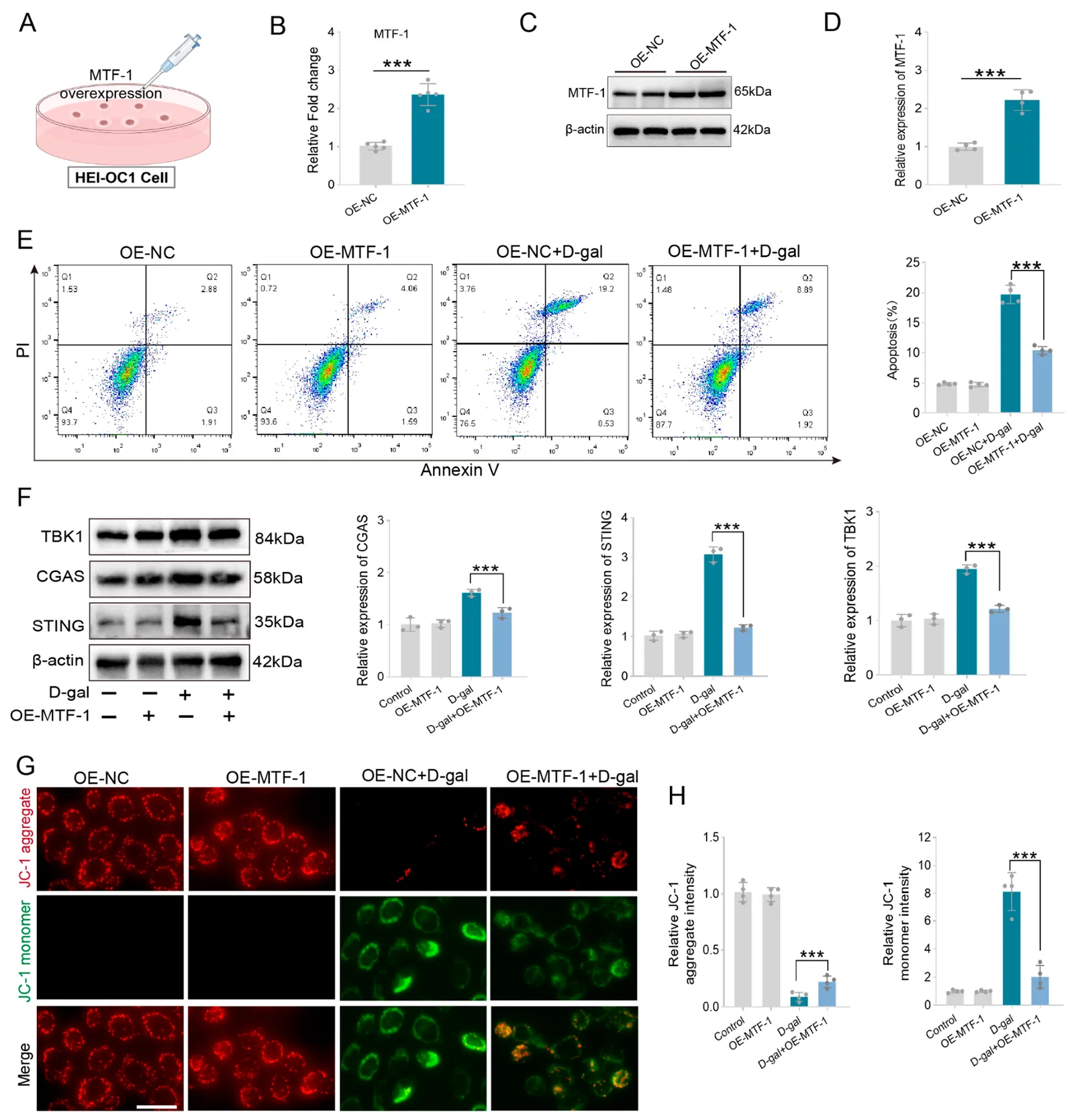
**Overexpression of MTF-1 significantly ameliorates D-gal induced cellular damage.** (**A**) Establishment of MTF-1 overexpression cell model. (**B**) Detection of MTF-1 mRNA expression levels by RT-PCR (*n* = 5 in each group). (**C**) Evaluation of MTF-1 protein levels by Western blot (*n* = 3 in each group). (**D**) Quantitative analysis of MTF-1 protein levels. (**E**) Flow cytometric analysis of the effect of MTF-1 overexpression on D-gal induced apoptosis (*n* = 5 in each group). (**F**) Representative Western blot images of TBK1, cGAS, and STING in different treatment groups (*n* = 3 in each group). (**G**) Assessment of mitochondrial membrane potential in different treatment groups using the JC-1 fluorescent probe (*n* = 5 in each group). (**H**) Quantitative analysis of JC-1 fluorescence intensity. *** *p* < 0.001. Scale bar (**G**) 40 μm.

## Data Availability

The original contributions presented in this study are included in the article. Further inquiries can be directed to the corresponding authors.
